# Analytical Assessment of the Antioxidant Properties of the Coneflower (*Echinacea purpurea* L. Moench) Grown with Various Mulch Materials

**DOI:** 10.3390/molecules29050971

**Published:** 2024-02-22

**Authors:** Celestina Adebimpe Ojo, Kinga Dziadek, Urszula Sadowska, Joanna Skoczylas, Aneta Kopeć

**Affiliations:** 1 Department of Human Nutrition and Dietetics, Faculty of Food Technology, University of Agriculture in Krakow, Balicka 122, 30-149 Krakow, Poland; celestina.adebimpe.ojo@student.urk.edu.pl (C.A.O.); kinga.dziadek@urk.edu.pl (K.D.); joannaskoczylas7@gmail.com (J.S.); 2 Faculty of Mechanisation and Energy Technologies in Agriculture, University of Agriculture in Krakow, Majora Łupaszki 6, 30-198 Krakow, Poland; urszula.sadowska@urk.edu.pl

**Keywords:** antioxidant properties, coneflower, chemical composition, *Echinacea purpurea*

## Abstract

Antioxidants are added to foods to decrease the adverse effect of reactive species that create undesirable compounds that destroy essential nutrients and, therefore, lower the nutritional, chemical and physical properties of foods. This study was carried out to determine the antioxidant properties of flowers and plant stems with leaves of *Echinacea purpurea* grown with mulches of different colours and thicknesses. Coneflowers were grown in the Experimental Station of the Agricultural University in Kraków, Poland. The mulching materials used were black, green and brown colours of 100 g/m^2^ and 80 g/m^2^ density. In plant material, e.g., flowers or plant stems plus leaves the proximate analysis, the total polyphenol content and the ability to scavenge free radicals (ABTS, DPPH and FRAP) were determined. The results show that flower samples had a higher content of compound proteins, ash and phenolic compounds. The mulching colour and density did not affect the proximate analysis of the *E. purpurea* plant. Based on the result of this study, *E. purpurea* is a potential source of natural antioxidants and can be used to improve the antioxidant activity of various food products as well as in cosmetics within the pharmaceutical industry.

## 1. Introduction

*Echinacea purpurea* L. (Moench) is a native and ornamental plant in the Atlantic geographical drainage area of the United States of America and Canada, but not including Mexico. The cultivation of this plant is also popular in Europe [1,2]. Based on some data in the literature, it is reported that *E. purpurea* is the last one in a list of the ten most frequently cultivated herbs in Poland [3,4].

*E. purpurea* is known as an “anti-infectious” agent because of an excellent potential to treat viral and bacterial infections and has been used to treat several infectious conditions ranging from simple acne and ulcers to mild septicemias [5,6,7]. *Echinacea purpurea* (*E. purpurea*) contains a number of chemical components including alkamides, polyalkenes, polyalkynes, chicoric and caftaric acids, as well as caffeic acid derivatives, glycoproteins and polysaccharides [4,8]. Some of the substances mentioned above exhibit an immunomodulatory effect [4,5]. *E. purpurea* volatile oil contains borneol, bornyl acetate, pentadeca-8-(Z)-en-2-one, germacrene D, caryophyllene and caryophyllene epoxide. Isobutyl amides of C11–C16 straight-chain fatty acids with olefinic or acetylenic bonds (or both) are found in the above parts of the *E.purpureae* herb, such as isomeric dodeca-(2E, 4E, 8Z, 10E/Z)-tetraenoic acid isobutyl amides, among others. The major active compound of the phenolic acid class found in the aerial parts is caffeic acid, with a concentration range of 1.2–3.1%. Chicoric acid methyl ester and other derivatives are also present [8]. Over the years, the immunomodulatory properties of *E. purpurea* have been reported, too. It was described that extract of *E. purpurea* considerably inhibited the growth of pathogenic yeasts such as *Candida albicans* [9,10,11] and bacteria (*Streptococcus pyogenes*, *Haemophilus influenzae* and *Legionella pneumophila*). It was also reported that the aqueous extract of *E. purpurea* has an antiviral activity for herpes simplex virus 1 (HSV-1) and herpes simplex virus 2 (HSV-2) [10,11,12].

According to the literature, *E. purpurea* requires high water capacity and well-oxygenated soil for root development [13]. Soil water deficit significantly inhibits plants’ growth and development, consequently reducing yield. Negative temperatures during winter are also a threat. Moreover, windy and low-temperature springs also limit plant growth during the initial growing season [4]. *E. purpurea* is one of Poland’s ten most frequently cultivated herbs [3,4] and, traditionally, Poland’s climatic conditions enable its cultivation throughout the country. However, in recent years, and due to global climate change, Polish agriculture has been negatively affected by uneven rainfall and drought periods, leading to weaker production [13,14].

Synthetic (inorganic) mulching material is frequently used in agriculture to protect crops from adverse environmental conditions including severe weather (low or high temperature, low rainfall), birds and insect crop damage [15]. However, mulching affects not only plant growth and yield but also plant chemical composition [16,17].

This study, therefore, aims to evaluate how different colours and thicknesses of synthetic mulching affect the chemical composition and antioxidant properties of *E. purpurea* flowers and leaves plus stems.

## 2. Results

### 2.1. Crude Protein Fat and Ash Content in Flowers, Plant Stems plus Leaves of E.purpurea

The obtained results indicated that flowers were richer in protein then the plant stems plus leaves (Table 1), showing that the different mulching materials with respect to colour did not significantly affect the protein content in *E. purpurea*. Among the flower samples, a significantly higher content of protein was assessed in samples F1 and F2; however, the lowest protein level was observed in sample F0. The result of the protein content in plant stems plus leaves showed the same trend as in the flower. Among this group, samples SL1 and SL2 were characterised by the highest content of protein, while sample SL0 was characterised by the lowest. Furthermore, it was observed that application at 100 g/m^2^ of mulch material in flower and plant stem plus leaves resulted in a higher protein content in *E. purpurea* irrespective of colour (Table 1) (the exceptions were the samples F3 and F6).

A comparison between sample F0 and SL0 showed that flowers were characterised by a higher crude fat level than plant stems plus leaves, with the exception of sample SL6 (Table 1). Among the flower samples, the highest content of fat was found in sample F0, compared to F1 and F5. Samples SL1, SL2, SL3 and SL5 showed the lowest fat content compared to the SL6 sample. Ash content in *E. purpurea* was richer in flowers than the plant stems plus leaves (Table 1). Among the flower samples, the highest content of ash was assessed in sample F1, while the lowest ash level was observed in sample F3. Between the plant stems plus leaves, samples SL2 and SL6 were characterised by a higher content of ash; therefore, the sample SL1 was characterised by the lowest. Different mulch applications with respect to colours and thicknesses did not significantly affect the ash content in *E. purpurea*.

### 2.2. Total Polyphenolic Content and Antioxidant Activity

The results obtained indicated that flowers were richer in total polyphenols and had higher antioxidant activity measured with all methods than plants stems plus leaves (Table 2). The different mulching materials with respect to thickness and colours did not significantly affect the total polyphenol content in *E. purpurea*. Among the flower samples, the significantly higher content of these compounds was assessed in samples F2, F4, F5 and F6, respectively. However, the lowest total polyphenol level was observed in sample F1. The result of total polyphenol content in plant stems plus leaves showed the same trend as the flower. Among this group, the highest content of total polyphenols was recorded in samples SL2 and SL4; therefore, sample SL1 showed the lowest value.

The results of the DPPH analysis revealed that flowers were characterised by higher antioxidant activity than the plant stems plus leaves (Table 3). Among the flower samples, the highest antioxidant properties were obtained in sample F4 compared to other flower samples. Variation was also observed in samples of plant stems plus leaves. In this group, the highest antioxidant activity was measured in sample SL4. However, samples SL1 and SL3 showed the lowest.

Among the flower samples, the highest antioxidant capacity, measured with the ABTS method, was obtained in sample F6, while the lowest was observed in sample F3. The result of an ABTS test carried out on the plant stems plus leaves indicated that samples SL0 and SL4 were characterised by significantly higher antioxidant activity; therefore, the samples SL1 and SL3 were characterised by the lowest (Table 2). Different mulching materials with respect to thickness significantly affected the antioxidant properties in *E. purpurea* flowers. The flowers of samples planted with 80 g/m^2^ of mulch material were characterised by significantly higher antioxidant activity compared to the flowers planted with 100 g/m^2^ of material.

The obtained results of a FRAP test indicated that flowers had higher antioxidant properties than plant stems plus leaves (Table 2). In the group of flowers, significantly, the highest antioxidant activity was assessed in samples F2 and F6. However, the lowest was observed in sample F1. Therefore, in the group of plant stems plus leaves, samples SL0 and SL4 were characterised by the highest antioxidant capacity; therefore, the samples SL1 and SL3 recorded as the lowest.

### 2.3. Polyphenolic Profile

The 25 types of polyphenols were detected by the HPLC method in the analysed samples, and their concentrations are shown in Table 3 and Table 4.

P-coumaric acid, chlorogenic acid and rutin were the dominant polyphenolic compounds in all samples, both in the flower as well as the plant stem plus leaves. Additionally, in the group of plant stems plus leaves, a high content of naringin was found. The obtained results showed that the flower samples had higher concentrations of almost all the polyphenol types in comparison with the plant stem plus leaf samples, regardless of the colour or thickness of the mulching material. The exceptions were hispidulin, which was identified only in plant stem plus leaf samples, as well as gallic acid, which was found in all samples in the group of plant stems plus leaves and only in samples F1 and F3 in the group of flower. From the result of the analysis of variance, a high significant variation in the concentration of polyphenol types was recorded within the different sample colours and weight of mulch applied at *p* < 0.05.

Furthermore, in flower samples that were planted with 100 g/m^2^ of mulching material (regardless of colour), a higher content of polyphenols was observed, such as sinapinic acid, catechin, apigenin and carnosol, in comparison with those planted with 80 g/m^2^ of mulching material. Therefore, a higher concentration of acacetin in flower samples as well as rutin and hesperidin in plant stems plus leaves samples was found in samples planted with 80 g/m^2^ of mulching material, regardless of colour, compared to those planted with 100 g/m^2^ of mulching material.

Additionally, the application of mulching material with a thickness of 80 g/m^2^ and a black and or brown colour resulted in an increase in the content of some polyphenolic compounds in flower samples (chlorogenic acid, 4-hydroxybenzoic acid, caffeic acid, p-coumaric acid and rutin) in comparison with using mulching material with a thickness of 100 g/m^2^ in the corresponding colours.

## 3. Discussion

The flowers of *E. purpurea* generally had higher protein content than the plant stems with leaves (Table 1). It can be also be suggested that mulch material used for cultivation had an effect on the content of protein and ash, especially in flowers. The use of black and green material during the cultivation of the plants resulted in a higher content of protein in the flowers and the mixture of the leaves and plant stems as compared to their controls. These changes can be explained by the protection of the soil and roots from dryness as well as changes in the temperature. These factors could synthesise protein. It was reported that the use of mulch material in the cultivation of various plants protects the evaporation of water from the soil; this increases water use efficiency, increases the soil temperature and nitrogen balance, as well as the yield of plants [15,18,19,20,21]. To the best of the authors’ knowledge of the currently available literature, there are no data concerning the content of crude protein, fat and ash in *E. purpurea’s* morphological parts cultivated in the traditional way or with mulching material. The composition of various types of extract from roots was evaluated for the bioactive substance content including phenolic acids, alkamides, polysaccharides and others [5,22,23].

In some studies, the proximate analysis was reported in various flowers that can be used as the herbs or ingredients of beverages. Rachkeeree et al. [24] reported that, in edible flowers of *Alpinia galanga* and *Hedychium forrestii*, the protein content was 33 g/100 g D.M. and 12.88 g/100 g D.M.. In the same flowers, the fat content was 11.9 g/100 g D.M. and 20.5 g/100 g D.M.. Li et al. [25] reported that the content of soluble protein and carbohydrates in peony flowers depended on the stage of the blooming flower. In chive flowers, the content of crude protein and crude fat was 18.5 g/100 g D.M. and 3.45 g/100 g D.M., respectively [26]. In our study, the variation observed in protein and ash content could be explained by many factors, involving the environment, abiotic stress, the genetic heritance maturity of the plants and the type of mulching material applied during the cultivation of the plants.

An important finding of this study is that the total polyphenol content and antioxidant activity of *E. Purpurea* flowers and plant stems plus leaves were affected by the mulch material used. It can be suggested that the mulch material could reduce soil aeration. Additionally, no mechanical weeding of the crop was performed, which also influenced the contact of air with soil. It could increase free radical production and increase the synthesis of phenolic compounds. According to data in the literature, polyphenols are involved in the stress protection of plants under harmful environmental conditions—not only drought, flooding and high or low temperature, but also ultraviolet radiation, salinity and heavy metal pollution. Their bioactivity and role in stress defence are generally attributed to their antioxidant activity. In addition, the different colours of the mulch determine changes in the intensity and absorption of radiation. Polyphenols also protect the plant against biotic stress. They are characterised by insecticidal/insect repellent activity and are a potential pest control strategy [27,28,29]. In this study, the content of total polyphenols in flower samples ranged from 9728.05 mg/100 g D.M. to 14,222 mg/100 g D.M. and in the combination of plant stems plus leaves ranged from 3256.16 mg/100g D.M. to 6395.19 mg/100 g D.M. The results of our study are in the range of the data published by Tsai et al. [30]. These authors measured the total phenolic content in flowers of *E. purpurea* in the range 9707 mg/100 g–39,982 mg/100 g. Chen et al. [31] reported that the level of phenolic compounds was 18,208 mg/100 g. Lower levels of total phenolic compounds than in our study were measured by Pellati et al. [32] in the roots of *E. purpurea* from Italy (2332 mg/100 g D.M.). These differences can be explained by the various morphological parts of the plant, climate condition and drying method. These authors used 40 °C for drying in the laboratory oven. In our study, we were using the freeze-dried method. In another study, the content of phenolic compound content in roots was measured in the range of 22.79 ± 0.37 mg/1 g [4,33].

Recent studies have shown that there is no universal method to evaluate antioxidant activity quantitatively and accurately [34]; therefore, the antioxidant activity of plants should be evaluated using several methods. Previous studies by Schlesier et al. [35] showed that when analysing antioxidant activity, it is preferable to use at least two methods. In this study, however, the analysis of the antioxidant activity of flowers and the combination of plant stems plus leaves of *E. purpurea* was performed using three methods: DPPH, ABTS and FRAP.

The antioxidant activity of flower samples, measured by the DPPH method in the present study for control samples, was 690.27 ± 40.75 µmol Trolox/1 g D.M. and that of the combination of plant stems with leaves was 351.93 ± 12.60 µmol Trolox/1 g D.M. It was reported that the flowers of *E. purpurea* have strong antioxidant activity [22,30,31].

Lower antioxidant activity, measured by the DPPH method, than in our study was reported in the leaves of *E. purpurea* (75.0 µmol Trolox/100 g D.M.) [36]. Hu and Kitts [22] reported that 0.8 mg/mL of the extract of *E. purpurea* scavenged in 20% DPPH radicals. Wojdyło et al. [36] reported lower antioxidant capacity, determined by the FRAP test, in the leaves of *E purpurea* (94.6 and 191.0 µM Trolox/100 g D.M., respectively), compared to our results.

The flower samples showed higher values of antioxidant activity compared to the combination of plant stems with leaves. Our data are different from the results reported by Ramazan et al. [1].

We have found that the major phenolic compounds in flowers and the mixture of plant stems with leaves were p-coumaric acid, chlorogenic acid, rutin, 4-hydroxybenzoic acid and hesperidin. Our results are different from data published by Tsali et al. [30], who reported that in flowers of *E. purpurea*, caffeic acid, chicoric acid and their derivatives were measured in higher amounts. A study carried out by Chen et al. [31] and Ramazan et al. [1] showed that cichoric acid was in the highest concentration in the leaves and flowers of *E. purpurea* extracted with methanol. Pellati et al. [33] reported that in roots, caftaric acid, chlorogenic acid and caffeic acid are the major polyphenolic compounds. In our study, p-coumaric acid was found in higher amounts in flowers and leaves. We have found that rutin was the major flavone in the evaluated plants (Table 4). What is more, the rutin content was higher in all the flower samples in comparison with the plant stem plus leaf samples. It has also been reported to be one of the polyphenolic compounds present in high concentration in medicinal plants and is responsible for the antioxidant capacity of plant extracts [37].

In this study, rutin concentration was the third highest concentration found in all the samples that were analysed, compared to other compounds. In general, flower samples showed more concentrations of almost all the polyphenolic compounds except for gallic acid, ferulic acid and sinapinic acid that showed more concentration in the plant stems plus leaves samples.

Based on the results of the antioxidant activity of *E. purpurea*, it is suggested that this plant can be used as a functional food (in the form of beverages or food additives). The high content of polyphenolic compounds means that *E. purpurea* extract may be helpful in the prevention and treatment of chronic non-communicable diseases such as obesity, atherosclerosis and other cardiovascular diseases, diabetes and cancer [38]. In addition, it can be suggested to use the whole extract due to the rich polyphenolic composition and the pleiotropic effects of polyphenols [39]. A single compound may not have as strong an effect as a mixture of them.

It can be suggested to producers to separate the flowers from the plan stems with leaves when harvesting the herb. The introduction of mulching material can also improve the yield of crops. Extracts obtained especially from the flowers can be used as a good source of antioxidants in food, used in the pharmaceutical or cosmetic industry. The dried flowers or/and plant stems plus leaves can also be used to prepare tea mixtures.

## 4. Material and Methods

### 4.1. Plant Material

**Figure 1:**
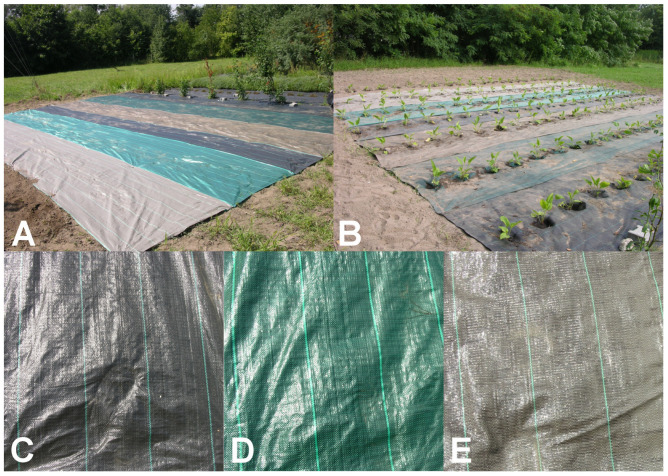
Mulching material. (**A**) Field covered with various types of mulching material prepared for cultivation. (**B**) *E purpureae* seedlings. (**C**) Example of black mulching material. (**D**) Example of green mulching material. (**E**) Example of brown mulching material (own source).

**Figure 2:**
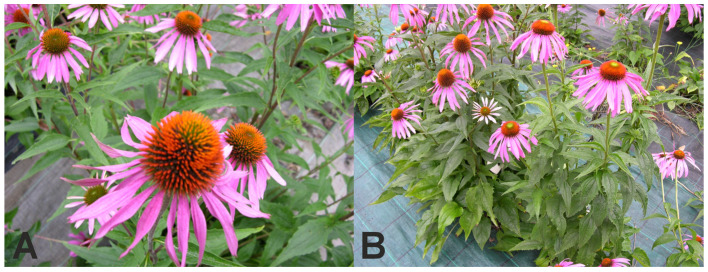
Mature *E. purpurea*. (**A**) Mature flowers. (**B**) Example of plants with the mulching material (own source).

Seedlings of *E. purpurea* were grown at the Experimental Station of the Agricultural University in Krakow, Poland, located in Mydlniki Kraków, (Poland). Synthetic mulching material was used to protect growing seedlings and avoid the use of herbicides. Synthetic mulching material was purchased in a local market. During cultivation, plants of *E. purpurea* and various types of mulch materials different colours (black, green and brown; thickness 100 g/m^2^ and 80 g/m^2^, respectively) were used (Table 5; Figure 1). The research materials were as follows. Samples of flower and combination of plant stems plus leaves were harvested two years after seedlings’ planting according to Farmacopea recommendations [40]. Samples were collected at the end of July during full blooming (Figure 2). In the laboratory, the flowers were separated from the plant stems plus leaves. The plants were washed to remove all impurities, e.g., soil and insects. Next, the samples were frozen and then lyophilised (Alpha 1–4 LS Cplus, Martin Christ Gefriertrocknungsanlagen GmbH, Osterodeam, Germany) for 24 h (capacitor temperature: −55 °C, vacuum: 1000 mbar). Lyophilised samples were ground and stored until the analyses.

### 4.2. Concentration of Protein, Ash and Crude Fat

In freeze-dried samples, the concentration of nitrogen was measured in accordance to the Kjeldahl method (AOAC no. 978.04) [41] as was previously described [42]. The percentage of total nitrogen was calculated following the formula N = (V × M × 14.007 × 100)/m, where N—total nitrogen content in g/100 g D.M. of the test research material [g/100 g D.M.]; V—volume of HCl used for titration of the sample [cm^3^]; M—molar concentration of HCl [mol/dm^3^]; 14.007—amount of nitrogen, which corresponds to 1 cm^3^ of HCl with a concentration of 1 mol/dm^3^; m—sample mass [g]. For the calculation of the protein, the conversion factor 6.25 was used. Crude fat content was determined with the Soxlet method in Soxtec Avanti’s 2050 Auto Extraction Unit (Tecator Foss, Hillerød, Sweden). For the extraction of crude fat petroleum, ether (POCh Gliwice, Poland) was used. Ash was determined in an electric muffle furnace (525 °C) (SNOL82.110, Utena, Lithuania).

### 4.3. Methanolic Extract Preparation

About 0.5 g of the ground plant material was weighed and mixed with an 80 cm^3^ solution of 70% methanol acidified with 0.1% of formic acid (*v*/*v*; POCh Giwice, Poland) and then placed in a shaker at room temperature for 2 h and protected from light. The mixture was centrifuged for 15 min (402× *g*, room temperature), and the supernatant was moved into a plastic container and stored at −22 °C [42].

### 4.4. Total Polyphenols Content and Antioxidant Activity

The content of total polyphenols in extracts of samples was determined using Folin–Ciocalteu reagent (Sigma-Aldrich, Saint Louis, MO, USA) [43]. The results are presented as the chlorogenic acid equivalent (CGA) in mg per 100 g of dry matter (D.M.) as was previously reported [43].

The antioxidant activity of methanolic extracts of coneflowers and leaves plus stems was determined as follows: ABTS (2.2′-Azino-bis (3-ethylbenzthiazoline-6-sulfonic acid)), FRAP (ferric-reducing antioxidant power) and DPPH (2,2-diphenyl-1-picrylhydrazyl). The determination of antioxidant activity using ABTS^•+^ free radicals was carried out according to the method described by Re et al. [44]. The stock solution was prepared by dissolving ABTS in water to a concentration of 7 mmol. The ABTS radical cation (ABTS^•+^) was prepared by reacting the ABTS solution with 2.45 mM potassium persulfate (final concentration) and allowing the mixture to stand for 16 h in the dark at room temperature before use. An ABTS^•+^ working solution was prepared by diluting the stock solution with 70% methanol to the absorbance of 0.740–0.750 at 734 nm. Next, 30 μL of the flower extract or 80 μL of the plant stem plus leaves extract, 970 μL or 920 μL of 70% methanol and then 2 mL of the ABTS^•+^ solution were added to a test tube. The mixture was stored in the dark at 30 °C for 6 min. The absorbance of the samples was determined at 734 nm (UV-1800, RayLeigh, Beijing Beifen Ruili Analytical Instrument Co., Ltd., Beijing, China). The FRAP assay was performed according to the Benzie and Strain [45] method. The working solution was prepared by mixing 100 mL of acetate buffer (pH 3.6), 10 mL of TPTZ solution (10 mmol/L TPTZ in 40 mmol/L HCl) and 10 mL of 20 mmol/L FeCl_3_·6H_2_O solution. Then, 30 μL of the flower extract or 80 μL of the plant stem plus leaf extract, 970 μL or 920 μL of 70% methanol and 3 mL of working solution of the FRAP reagent were transferred into a test tube. The mixture was stored in a dark at room temperature for 10 min. The absorbance of the samples was assessed at 593 nm (UV-1800, RayLeigh, Beijing Beifen Ruili Analytical Instrument Co., Ltd., Beijing, China). The determination of antioxidant activity with the DPPH method was conducted in accordance to Miliauskas et al. [46]. The stock solution was prepared by dissolving 5 mg of DPPH in 100 mL of methanol. The DPPH^•^ working solution was obtained by diluting the stock solution with methanol to the absorbance of 0.900–1.000 at 515 nm. Next, 50 μL of the flower or the plant stem plus leaf extract was added to 1450 μL of methanol and 3 mL of FRAP working solution; all were added into a test tube. The mixture was stored in the dark at room temperature for 10 min. All results were compared to the concentration–response curve of the standard Trolox solution and presented as µmol of Trolox equivalent per g of D.M. (TEAC) of samples. For the ABTS and DPPH assays, the range of Trolox concentrations was 1.95–62.5 µmol Trolox/L; however, for the FRAP assay, it was 31.25–250 µmol Trolox/L.

### 4.5. Polyphenols Profile

For HPLC polyphenolic compound analysis, acidified methanolic extracts were used to determine polyphenolic compounds with the HPLC method using the Prominence-i LC 2030C D3 Plus system (Shimadzu, Kyoto, Japan) along with a DAD detector and a Luna Omega 5 µm Polar C18, 100 A, 250 × 10 mm Phenomenex column (Torrance, CA, USA). The separation of phenolic compounds was performed at 40 °C. The mobile phase was a mixture of eluents: A, 0.1% formic acid in water (*v*/*v*), and B, 0.1% formic acid in methanol (*v*/*v*). The mobile phase flow rate was 1.2 mL/min. The analysis was carried out with the following gradient conditions: from 20% to 40% B in 10 min, 40% B for 10 min, from 40% to 50% B in 10 min, from 50% to 60% B in 5 min, 60% B for 5 min, from 60 to 70% B in 5 min, from 70% to 90% B in 5 min, 90% B for 5 min, from 90% to 20% B (the initial condition) in 1 min, and 20% B for 4 min. This resulted in a total run time of 60 min. The injection volume of tested samples was 20 μL. A stock standard solution (100 mg/L) of each polyphenolic compound was prepared in 70% methanol acidified methanol (0.1% formic acid (POCH, Gliwice, Poland). The identification of compounds was made on the basis of retention times. The calibration curves of the polyphenol standards were made by the dilution of stock standard solutions in 0.1% formic acid in 70% methanol (*v*/*v*). The range of the calibration curve was 0.25–4.0 mg/L. All the solutions were filtered through a 0.22 µm filter as was previously reported [47].

The following polyphenolic compounds were determined based on standards: gallic acid, catechin, chlorogenic acid, 4-hydroxybenzoic acid, epicatechin, caffeic acid, vanillic acid, syringic acid, p-coumaric acid, ferulic acid, sinapinic acid, naringin, rutin, hesperidin, rosmarinic acid, myricetin, quercetin, luteolin, kaempferol, apigenin, isorhamnetin, hispidulin, acacetin, carnosol and carnosic acid (Sigma, Saint Louis, MO, USA). Measurements were based on LabSolution ver. 5.93 from Shimadzu Corporation (Kyoto, Japan). The levels of quercetin, luteolin, apigenin and myricetin were determined only in their free aglycone form.

### 4.6. Statistical Analysis

The analyses were performed in two or three parallel replications. Multiple-way analysis of variance was also carried out using the program Statistica version 13.1, Dell Inc., Tulsa, OK, USA, 2016. Significant differences were assessed using Duncan’s test (*p* ≤ 0.05).

## 5. Conclusions

Worldwide, edible flowers are gaining increased interest among consumers. The results of this study indicate that the flower and plant stem plus leaves of *E. purpurea* are rich sources of polyphenols and exhibit high antioxidant activity, making it a potential functional food. The extract of *E. purpurea*, especially the flower extract, can be helpful in the prevention and treatment of chronic non-communicable diseases. Additionally, under favourable metrological conditions, it is possible to harvest them twice in one year. The introduction of mulching material can also improve the yield of crops. As a source of phytochemicals, it opens up new possibilities for the creation of new functional foods, such as beverages or food additives (lyophilised plant or powdered extract). On the other hand, there is a need for further research on *E. purpurea*, in particular, in vivo studies to confirm beneficial effects in the organism, determine the dose and check the safety of use and possible side effects.

## Figures and Tables

**Table 1:** Protein, crude fat and ash content in the flower and the mixture of plant stems plus leaves of *E. purpurea* [g/100 g D.M.].

Sample Name	Protein	Crude Fat	Ash
F0	13.21 ± 0.11 ^de^	1.99 ± 0.13 ^c^	8.59 ± 0.06 ^g^
F1	17.10 ± 0.89 ^fg^	1.02 ± 0.25 ^ab^	9.09 ± 0.65 ^h^
F2	17.47 ± 0.08 ^g^	1.23 ± 0.04 ^bc^	8.32 ± 0.07 ^efg^
F3	15.40 ± 0.48 ^e^	1.45 ± 0.23 ^bc^	7.74 ± 0.08 ^d^
F4	15.58 ± 0.01 ^e^	1.47 ± 0.38 ^bc^	8.39 ± 0.05 ^efg^
F5	15.98 ± 0.23 ^ef^	0.64 ± 0.04 ^a^	7.87 ± 0.15 ^de^
F6	15.67 ± 0.38 ^ef^	1.67 ± 0.00 ^bc^	8.05 ± 0.11 ^def^
SL0	7.20 ± 0.76 ^a^	0.85 ± 0.11 ^ab^	6.99 ± 0.37 ^c^
SL1	12.00 ± 0.01 ^de^	0.60 ± 0.02 ^a^	5.45 ± 0.10 ^a^
SL2	12.04 ± 1.68 ^de^	0.62 ± 0.08 ^a^	8.36 ± 0.00 ^efg^
SL3	9.34 ± 0.86 ^b^	0.60 ± 0.03 ^a^	6.25 ± 0.02 ^b^
SL4	11.38 ± 0.27 ^cd^	1.25 ± 0.04 ^abc^	7.91 ± 0.09 ^de^
SL5	8.90 ± 0.42 ^b^	0.73 ± 0.11 ^a^	6.04 ± 0.28 ^b^
SL6	10.12 ± 0.24 ^bc^	2.06 ± 0.15 ^c^	8.51 ± 0.02 ^fg^
Part of the plant			
Flower	15.77 ± 3.94 ^B^	1.35 ± 0.56 ^A^	8.29 ± 0.48 ^B^
Stems plus leaves	10.14 ± 1.83 ^A^	0.96 ± 0.52 ^A^	7.07 ± 1.17 ^A^
*p*-value	0.001	0.065	0.001
Mulching material colour			
Control	10.20 ± 3.50 ^A^	1.42 ± 0.67 ^A^	7.79 ± 0.95 ^A^
Black	14.02 ± 2.59 ^A^	1.09 ± 0.39 ^A^	7.71 ± 1.49 ^A^
Green	13.60 ± 3.65 ^A^	0.80 ± 0.27 ^A^	7.65 ± 1.02 ^A^
Brown	12.63 ± 3.15 ^A^	1.45 ± 0.74 ^A^	7.64 ± 0.91 ^A^
*p*-value	0.264	0.097	0.996
Thickness of mulching material g/100 m^2^			
Control	10.20 ± 3.50 ^A^	1.42 ± 0.67 ^A^	7.79 ± 0.95 ^A^
100	13.89 ± 3.18 ^A^	0.92 ± 0.52 ^A^	7.53 ± 1.35 ^A^
80	12.94 ± 3.03 ^A^	1.31 ± 0.54 ^A^	7.795 ± 0.86 ^A^
*p*-value	0.150	0.153	0.827

Mean values in the same column with different letters (a–h) are statistically different (*p* < 0.05); mean values with different capital letters (A,B) in the same column are statistically different; differences are between the experiment factors, e.g., the part of the plant, colour of the mulching material, and thickness of the mulching material; results are expressed as mean ± SD; D.M., dry matter; F0, flower control (cultivated without mulching material); F1, flower cultivated with 100 g/m^2^ black mulching material; F2, flower cultivated with 100 g/m^2^ green mulching material; F3, flower cultivated with 100 g/m^2^ brown mulching material; F4, flower cultivated with 80 g/m^2^ black mulching material; F5, flower cultivated with 80 g/m^2^ green mulching material; F6, flower cultivated with 80 g/m^2^ brown mulching material; SL0, plant stems plus leaves—control (cultivated with no mulching material); SL1, plant stems plus leaves cultivated with 100 g/m^2^ black mulching material, SL2, plant stems plus leaves cultivated with 100 g/m^2^ green mulching material, SL3, plant stems plus leaves cultivated with 100 g/m^2^ brown mulching material; SL4, plant stems plus leaves cultivated with 80 g/m^2^ black mulching material; SL5, plant stems plus leaves planted with 80 g/m^2^ green mulching material; SL6, plant stems plus leaves planted with 80 g/m^2^ brown mulching material.

**Table 2:** Total polyphenol concentration and antioxidant activity of flowers and a mixture of plant stems plus leaves of coneflower.

Sample Name	Total Polyphenols (mg/100 g D.M.) *	DPPH (µmol Trolox/1 g D.M.)	ABTS (µmol Trolox/1 g D.M.)	FRAP (µmol Trolox/1 g D.M.)
F0	12,881.59 ± 547.89 ^g^	690.27 ± 40.75 ^f^	547.70 ± 17.09 ^h^	1680.71 ± 73.22 ^f^
F1	9728.05 ± 232.05 ^e^	609.74 ± 27.52 ^e^	356.36 ± 7.99 ^f^	1501.03 ± 12.05 ^e^
F2	14,222.89 ± 233.76 ^h^	860.06 ± 10.60 ^h^	331.66 ± 31.55 ^ef^	2319.29 ± 23.52 ^i^
F3	10,821.37 ± 182.08 ^f^	660.21 ± 27.52 ^ef^	314.67 ± 9.13 ^e^	2141.01 ± 11.63 ^g^
F4	13,941.18 ± 0.00 ^h^	937.17 ± 30.80 ^i^	512.66 ± 56.92 ^g^	2220.66 ± 67.23 ^h^
F5	13,663.35 ± 441.41 ^h^	824.41 ± 18.58 ^h^	511.51 ± 23.60 ^gh^	2116.97 ± 26.34 ^g^
F6	14,129.95 ± 156.91 ^h^	752.51 ± 29.29 ^g^	588.66 ± 12.06 ^i^	2294.97 ± 33.79 ^i^
SL0	5363.62 ± 70.56 ^c^	351.93 ± 12.60 ^bc^	189.46 ± 18.46 ^d^	798.95 ± 6.99 ^d^
SL1	3256.16 ± 26.52 ^a^	269.36 ± 1.40 ^a^	45.63 ± 4.47 ^a^	438.78 ± 20.28 ^a^
SL2	6395.19 ± 7.91 ^d^	373.40 ± 37.35 ^c^	109.76 ± 14.32 ^b^	595.36 ± 4.46 ^c^
SL3	4270.80 ± 54.41 ^b^	268.23 ± 4.32 ^a^	45.92 ± 6.56 ^a^	457.23 ± 3.85 ^a^
SL4	6673.99 ± 75.66 ^d^	432.54 ± 28.51 ^d^	201.02 ± 9.04 ^d^	779.71 ± 8.88 ^d^
SL5	4257.47 ± 425.34 ^b^	316.69 ± 9.00 ^ab^	80.46 ± 5.92 ^ab^	515.39 ± 11.72 ^b^
SL6	4843.00 ± 25.34 ^c^	389.09 ± 8.04 ^cd^	151.97 ± 5.72 ^c^	602.70 ± 8.72 ^c^
		Part of plant		
flower	12,770 ± 1706 ^B^	762 ± 115 ^B^	452 ± 110 ^B^	2039 ± 305 ^B^
Steams plus leaves	5009 ± 1168 ^A^	343 ± 60 ^A^	118 ± 62 ^A^	598 ± 137 ^A^
*p*-value	<0.001	<0.001	<0.001	<0.001
Colour of mulching material				
Control	9123 ± 4125 ^A^	521 ± 197 ^A^	368 ± 197 ^A^	1239 ± 473 ^A^
Black	8399 ± 4109 ^A^	562 ± 265 ^A^	279 ± 177 ^A^	1235 ± 747 ^A^
Green	9634 ± 4578 ^A^	593 ± 267 ^A^	258 ± 184 ^A^	1386 ± 872 ^A^
brown	8516 ± 4317 ^A^	517 ± 210 ^A^	275 ± 214 ^A^	1374 ± 885 ^A^
*p*-value	0.580	0.554	0.041	0.761
		Thickness g/m^2^		
control	9122 ± 4125 ^A^	521 ± 199 ^A^	368 ± 197 ^B^	1239 ± 473 ^A^
100	8116 ± 3955 ^A^	507 ± 230 ^A^	201 ± 140 ^A^	1242 ± 810 ^A^
80	9585 ± 4519 ^A^	609 ± 249 ^A^	341 ± 208 ^AB^	1422 ± 818 ^A^
*p*-value	0.580	0.554	0.041	0.761

Mean values in the same column with different letters (a–i) are statistically different (*p* < 0.05); mean values with different capital letters (A,B) in the same column are statistically different; differences are between the experiment factors, e.g., the part of the plant, the colour of the mulching material and the thickness of the mulching material; results are expressed as mean ± SD; D.M., dry matter; F0, flower control (cultivated without mulching material); F1, flower cultivated with 100g/m^2^ black mulching material; F2, flower cultivated with 100 g/m^2^ green mulching material; F3, flower cultivated with 100 g/m^2^ brown mulching material; F4, flower cultivated with 80 g/m^2^ black mulching material; F5, flower cultivated with 80 g/m^2^ green mulching material; F6, flower cultivated with 80 g/m^2^ brown mulching material; SL0, stems plus leaves—control (cultivated without mulching material); SL1, plant stems plus leaves cultivated with 100 g/m^2^ black mulching material; SL2, plant stems plus leaves cultivated with 100 g/m^2^ green mulching material, SL3, plant stems plus leaves cultivated with 100 g/m^2^brown mulching material; SL4, plant stems plus leaves cultivated with 80 g/m^2^ black mulching material; SL5, plant stems plus leaves planted with 80 g/m^2^ green mulching material; SL6, plant stems plus leaves planted with 80 g/m^2^ brown mulching material; * chlorogenic acid equivalent.

**Table 3:** The profile of phenolic acids in flowers and a mixture of stems plus leaves of coneflower [mg/100 g D.M.].

Sample	Gallic Acid	Chlorogenic Acid	4 Hydroxybenzoic Acid	Caffeic Acid	Vanillic Acid	Syringic Acid	p-Coumaric Acid	Ferulic Acid	Sinapinic Acid mg/100 g	Rosmarinic Acid mg/100 g	Carnosic Acid * mg/100 g
F0	nd	276 ± 0.03 ^i^	54.46 ± 0.04 ^h^	14.42 ± 1.66 ^e^	3.03 ± 0.01 ^cd^	13.30 ± 0.09 ^h^	1719 ± 1.58 ^j^	2.83 ± 0.10 ^a^	51.64 ± 1.36 ^c^	3.91 ± 0.09 ^c^	91.05 ± 0.03 ^f^
F1	2.53 ± 0.3 ^b^	195 ± 0.25 ^e^	40.79 ± 0.11 ^e^	16.68 ± 0.07 ^f^	9.46 ± 1.36 ^i^	7.96 ± 0.04 ^g^	1354 ± 2.17 ^h^	2.63 ± 0.03 ^a^	50.20 ± 1.46 ^c^	2.10 ± 0.03 ^ab^	43.61 ± 0.69 ^c^
F2	nd	331 ± 0.07 ^k^	63.64 ± 1.06 ^j^	31.22 ± 0.04 ^j^	13.83 ± 0.09 ^j^	4.57 ± 0.17 ^bc^	2053 ± 7.38 ^m^	3.72 ± 0.15 ^b^	52.71 ± 1.16 ^c^	2.60 ± 0.06 ^b^	67.30 ± 0.73 ^de^
F3	5.27 ± 0.01 ^f^	187 ± 0.06 ^d^	37.45 ± 0.04 ^d^	14.26 ± 0.91 ^e^	6.07 ± 0.01 ^g^	3.76 ± 0.20 ^ab^	1385 ± 4.08 ^i^	nd	40.59 ± 0.61 ^ab^	1.95 ± 0.03 ^a^	70.89 ± 0.22 ^e^
F4	nd	358 ± 0.24 ^l^	58.78 ± 0.03 ^i^	21.00 ± 0.00 ^g^	5.28 ± 0.03 ^f^	2.83 ± 1.06 ^a^	1934 ± 7.53 ^k^	nd	42.61 ± 0.24 ^b^	20.90 ± 0.56 ^g^	38.63 ± 0.26 ^c^
F5	nd	230 ± 6.56 ^h^	47.33 ± 1.39 ^f^	25.33 ± 0.00 ^i^	8.01 ± 0.02 ^h^	13.56 ± 1.28 ^h^	2035 ± 1.31 ^l^	nd	38.28 ± 0.54 ^a^	14.30 ± 0.09 ^e^	61.31 ± 14.73 ^d^
F6	nd	221 ± 0.94 ^g^	41.40 ± 0.05 ^e^	22.29 ± 0.45 ^h^	6.59 ± 0.02 ^g^	16.43 ± 0.29 ^i^	2107 ± 4.45 ^n^	nd	39.58 ± 0.29 ^a^	1.60 ± 0.03 ^a^	106.44 ± 0.17 ^g^
SL0	2.50 ± 0.01 ^b^	282 ± 0.33 ^j^	54.34 ± 0.09 ^h^	8.31 ± 0.04 ^d^	3.00 ± 0.01 ^cd^	14.10 ± 0.15 ^h^	470 ± 0.86 ^f^	nd	100.35 ± 0.65 ^g^	13.90 ± 0.46 ^e^	2.75 ± 0.16 ^a^
SL1	2.54 ± 0.01 ^b^	86 ± 0.09 ^b^	17.04 ± 0.03 ^b^	5.58 ± 0.03 ^ab^	1.64 ± 0.01 ^ab^	5.23 ± 0.10 ^cd^	318 ± 2.85 ^c^	nd	69.39 ± 1.54 ^d^	5.66 ± 0.09 ^d^	7.30 ± 0.63 ^ab^
SL2	3.00 ± 0.07 ^d^	202 ± 0.05 ^f^	40.67 ± 0.11 ^e^	7.27 ± 0.02 ^cd^	4.30 ± 0.10 ^e^	5.62 ± 0.03 ^de^	422 ± 3.55 ^e^	67.96 ± 0.16 ^d^	99.81 ± 1.99 ^g^	5.34 ± 0.07 ^d^	7.13 ± 0.11 ^ab^
SL3	2.87 ± 0.02 ^c^	197.66 ± 1.09 ^e^	37.35 ± 0.02 ^d^	4.47 ± 0.00 ^a^	1.41 ± 0.02 ^a^	6.77 ± 0.00 ^f^	203 ± 2.01 ^a^	41.78 ± 0.27 ^c^	51.41 ± 0.88 ^c^	3.89 ± 0.06 ^c^	8.69 ± 0.38 ^ab^
SL4	3.60 ± 0.11 ^e^	274.60 ± 0.14 ^i^	49.76 ± 0.10 ^g^	6.52 ± 0.03 ^bc^	4.65 ± 0.08 ^ef^	6.46 ± 0.02 ^ef^	548 ± 3.93 ^g^	96.14 ± 0.14 ^e^	120.44 ± 1.08 ^h^	22.83 ± 0.51 ^h^	12.86 ± 0.89 ^b^
SL5	5.53 ± 0.09 ^g^	70.19 ± 0.16 ^a^	13.27 ± 0.02 ^a^	5.24 ± 0.03 ^a^	3.24 ± 0.17 ^d^	4.53 ± 0.02 ^bc^	309 ± 2.04 ^b^	nd	76.15 ± 0.03 ^e^	1.90 ± 0.03 ^a^	6.30 ± 0.62 ^ab^
SL6	2.10 ± 0.00 ^a^	109.20 ± 0.16 ^c^	19.84 ± 0.01 ^c^	6.82 ± 0.06 ^c^	2.30 ± 0.00 ^bc^	7.49 ± 0.16 ^fg^	360 ± 2.94 ^d^	nd	83.93 ± 1.51 ^f^	18.41 ± 0.33 ^f^	8.06 ± 1.08 ^ab^

Mean values in the same column with different letters (a–n) are statistically different (*p* < 0.05); results are expressed as mean ± SD; D.M., dry matter; * phenolic diterpene; F0, flower control (cultivated without mulching material); F1, flower cultivated with 100 g/m^2^ black mulching material; F2, flower cultivated with 100 g/m^2^ green mulching material; F3, flower cultivated with 100 g/m^2^ brown mulching material; F4, flower cultivated with 80 g/m^2^ black mulching material; F5, flower cultivated with 80 g/m^2^ green mulching material; F6, flower cultivated with 80 g/m^2^ brown mulching material; SL0, plant stems plus leaves—control (cultivated without mulching material); SL1, plant stems plus leaves cultivated with 100 g/m^2^ black mulching material, SL2, plant stems plus leaves cultivated with 100 g/m^2^ green mulching material; SL3, plant stems plus leaves cultivated with 100 g/m^2^ brown mulching material; SL4, plant stems plus leaves cultivated with 80 g/m^2^ black mulching material; SL5, plant stems plus leaves planted with 80 g/m^2^ green mulching material; SL6, plant stems plus leaves planted with 80 g/m^2^ brown mulching material; nd—not identified.

**Table 4:** The profile of polyphenols in flowers and a mixture of stems plus leaves of coneflower [mg/100 g D.M.].

Sample Name	Catechin	Epicatechin	Naringin	Rutin	Hesperidin	Myricetin	Luteolin	Kaempferol	Apigenin	Hispidulin	Acacetin	Carnosol *
F0	20.10 ± 4.65 ^b^	29.10 ± 5.56 ^ef^	3.79 ± 0.09 ^a^	119 ± 0.9 ^g^	53.58 ± 0.11 ^e^	15.57 ± 0.00 ^c^	1.89 ± 0.00 ^c^	2.11 ± 0.00 ^a^	3.05 ± 0.01 ^b^	nd	11.17 ± 0.03 ^i^	11.26 ± 0.19 ^ab^
F1	24.06 ± 7.16 ^bc^	32.34 ± 10.41 ^f^	35.58 ± 1.33 ^b^	203 ± 0.01 ^i^	86.24 ± 0.17 ^h^	28.50 ± 0.07 ^f^	nd	2.68 ± 0.01 ^e^	3.98 ± 0.24 ^e^	nd	4.32 ± 0.00 ^d^	24.23 ± 24.74 ^abc^
F2	30.18 ± 0.13 ^c^	23.92 ± 0.07 ^bcde^	115 ± 1.83 ^e^	257 ± 1.14 ^m^	107. ± 0.21 ^l^	31.69 ± 0.11 ^h^	2.44 ± 0.00 ^f^	2.60 ± 0.02 ^d^	3.67 ± 0.00 ^d^	nd	6.06 ± 0.04 ^f^	31.47 ± 31.71 ^abc^
F3	25.34 ± 0.10 ^bc^	28.88 ± 0.00 ^def^	154 ± 4.60 ^g^	177 ± 0.64 ^h^	103 ± 1.83 ^k^	22.57 ± 0.03 ^e^	nd	3.41 ± 0.03 ^f^	5.68 ± 0.01 ^f^	nd	6.61 ± 0.03 ^g^	8.64 ± 0.16 ^a^
F4	6.23 ± 0.26 ^a^	20.71 ± 0.48 ^abcd^	197 ± 2.74 ^h^	217 ± 0.89 ^l^	98.16 ± 0.70 ^j^	38.77 ± 1.68 ^i^	1.97 ± 0.02 ^d^	2.23 ± 0.00 ^b^	3.15 ± 0.00 ^bc^	nd	4.72 ± 0.00 ^e^	8.37 ± 0.17 ^a^
F5	3.53 ± 4.99 ^a^	23.88 ± 5.31 ^bcde^	14.83 ± 19.19 ^a^	215 ± 0.54 ^k^	87.87 ± 0.06 ^i^	30.09 ± 0.12 ^g^	2.02 ± 0.05 ^d^	2.62 ± 0.00 ^d^	1.45 ± 0.03 ^a^	nd	10.55 ± 0.05 ^h^	7.22 ± 0.14 ^a^
F6	3.84 ± 0.39 ^a^	14.36 ± 0.19 ^a^	3.05 ± 0.08 ^a^	210 ± 1.11 ^j^	76.98 ± 0.03 ^g^	21.37 ± 0.02 ^d^	1.69 ± 0.02 ^a^	2.44 ± 0.02 ^c^	3.33 ± 0.03 ^c^	nd	14.68 ± 0.05 ^j^	7.96 ± 0.03 ^a^
SL0	nd	27.01 ± 0.18 ^cdef^	134 ± 0.95 ^f^	94.36 ± 0.49 ^f^	66.56 ± 0.64 ^f^	2.93 ± 0.06 ^b^	1.79 ± 0.07 ^b^	nd	nd	1.67 ± 0.00 ^a^	nd	26.07 ± 23.96 ^abc^
SL1	7.78 ± 0.49 ^a^	19.91 ± 0.25 ^abc^	56 ± 0.43 ^c^	42.95 ± 0.06 ^a^	26.21 ± 0.12 ^a^	nd	nd	nd	nd	1.71 ± 0.00 ^a^	nd	43.38 ± 0.13 ^c^
SL2	10.11 ± 0.70 ^a^	13.94 ± 0.03 ^a^	129 ± 1.70 ^f^	58.78 ± 0.36 ^c^	42.58 ± 0.46 ^c^	1.63 ± 0.02 ^a^	2.16 ± 0.02 ^e^	nd	nd	nd	nd	46.30 ± 0.00 ^c^
SL3	48.39 ± 5.85 ^d^	15.77 ± 0.39 ^ab^	89 ± 0.33 ^d^	54.40 ± 0.02 ^b^	32.28 ± 0.56 ^b^	1.86 ± 0.02 ^ab^	nd	nd	nd	1.93 ± 0.00 ^b^	1.64 ± 0.02 ^a^	43.27 ± 0.43 ^c^
SL4	nd	19.60 ± 0.06 ^abc^	187 ± 15.97 ^h^	83.73 ± 1.12 ^e^	53.98 ± 0.11 ^e^	1.98 ± 0.02 ^ab^	nd	nd	nd	2.49 ± 0.00 ^c^	1.94 ± 0.02 ^b^	45.79 ± 0.44 ^c^
SL5	nd	19.47 ± 0.33 ^abc^	63. ± 0.21 ^c^	84.13 ± 0.21 ^e^	47.43 ± 0.33 ^d^	2.21 ± 0.05 ^ab^	nd	2.45 ± 0.02 ^c^	nd	1.94 ± 0.05 ^b^	nd	45.68 ± 0.28 ^c^
SL6	nd	31.57 ± 0.65 ^ef^	89. ± 0.08 ^d^	78.94 ± 0.25 ^d^	48.44 ± 0.33 ^d^	2.11 ± 0.13 ^ab^	nd	nd	1.56 ± 0.00 ^a^	nd	2.33 ± 0.01 ^c^	39.33 ± 0.25 ^bc^

Mean values in the same column with different letters (a–m) are statistically different (*p* < 0.05); results are expressed as mean ± SD; D.M., dry matter; * phenolic diterpene; F0, flower control (cultivated without mulching material); F1, flower cultivated with 100 g/m^2^ black mulching material; F2, flower cultivated with 100 g/m^2^ green mulching material; F3, flower cultivated with 100 g/m^2^ brown mulching material; F4, flower cultivated with 80 g/m^2^ black mulching material; F5, flower cultivated with 80 g/m^2^ green mulching material; F6, flower cultivated with 80 g/m^2^ brown mulching material; SL0, plant stems plus leaves—control (cultivated with no mulching material); SL1, plant stems plus brown mulching material; SL4, plant stems plus leaves cultivated with 80 g/m^2^ black mulching material; SL5, plant stems plus leaves planted with 80 g/m^2^ green mulching material; SL6, plant stems plus leaves planted with 80 g/m^2^brown mulching material; nd—not identified.

**Table 5:** *E. purpurea* treatment codes.

Code	Treatment
F0	Flower/control
F1	Flower/black mulching; density 100 g/m^2^
F2	Flower/green mulching; density 100 g/m^2^
F3	Flower/brown mulching; density 100 g/m^2^
F4	Flower/black mulching; density 80 g/m^2^
F5	Flower/green mulching; density 80 g/m^2^
F6	Flower brown mulching; density 80 g/m^2^
SL0	Stem plus leaves control
SL1	Stem plus leaves/black mulching; density 100 g/m^2^
SL2	Stem plus leaves/green mulching; density 100 g/m^2^
SL3	Stem plus leaves/brown mulching; density 100 g/m^2^
SL4	Stem plus leaves/black mulching; density 80 g/m^2^
SL5	Stem plus leaves/green mulching; density 80 g/m^2^
SL6	Stem plus leaves/brown mulching; density 80 g/m^2^

## Data Availability

The data presented in this study are available on request from the corresponding author.
